# 6-Chloro-9-(2-nitro­phenyl­sulfon­yl)-9*H*-purine

**DOI:** 10.1107/S1600536811003102

**Published:** 2011-02-23

**Authors:** Ning-Yu Wang, Mei Deng, Yong Xia, Luo-Ting Yu

**Affiliations:** a State Key Laboratory of Biotherapy and Cancer Center, West China Hospital, West China Medical School, Sichuan University, Chengdu 610041, People’s Republic of China

## Abstract

The title compound, C_11_H_6_ClN_5_O_4_S, crystallized with two independent mol­ecules in the asymmetric unit. The benzene ring makes dihedral angles of 66.46 (8) and 85.77 (9)° with the mean plane of the purine ring in the two mol­ecules. In the crystal, inter­molecular π–π stacking inter­actions [centroid–centroid distance = 3.8968 (12) Å], C—Cl⋯π inter­actions [Cl⋯centroid = 3.2505 (10) Å, C—Cl⋯centroid = 161.56 (18)°] and non-classical C—H⋯O and C—H⋯N hydrogen bonds link the molecules.

## Related literature

For general background to the chemistry, biological activity and applications of purine derivatives, see: Scozzafava *et al.* (2001 ▶); Bakkestuen *et al.* (2005 ▶).
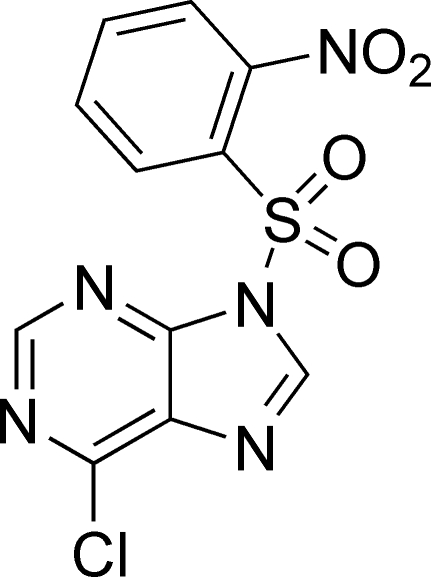


## Experimental

### 

#### Crystal data

C_11_H_6_ClN_5_O_4_S
*M* *_r_* = 339.72
Triclinic, 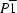

*a* = 10.0055 (3) Å
*b* = 10.6931 (5) Å
*c* = 12.5378 (5) Å
α = 93.692 (3)°
β = 97.136 (3)°
γ = 93.995 (3)°
*V* = 1324.16 (9) Å^3^
*Z* = 4
Mo *K*α radiation
μ = 0.47 mm^−1^
*T* = 293 K
0.42 × 0.40 × 0.35 mm

#### Data collection

Oxford Diffraction Xcalibur Eos diffractometer
Absorption correction: multi-scan (*CrysAlis PRO*; Oxford Diffraction, 2006 ▶) *T*_min_ = 0.992, *T*_max_ = 1.0
10984 measured reflections
5403 independent reflections
4389 reflections with *I* > 2σ(*I*)
*R*_int_ = 0.018

#### Refinement

*R*[*F* ^2^ > 2σ(*F* ^2^)] = 0.037
*wR*(*F* ^2^) = 0.092
*S* = 1.02
5403 reflections
397 parameters
H-atom parameters constrained
Δρ_max_ = 0.29 e Å^−3^
Δρ_min_ = −0.38 e Å^−3^

### 

Data collection: *CrysAlis PRO* (Oxford Diffraction, 2006 ▶); cell refinement: *CrysAlis PRO*; data reduction: *CrysAlis PRO*; program(s) used to solve structure: *SHELXS97* (Sheldrick, 2008 ▶); program(s) used to refine structure: *SHELXL97* (Sheldrick, 2008 ▶); molecular graphics: *OLEX2* (Dolomanov *et al.*, 2009 ▶) and *Mercury* (Macrae *et al.*, 2006 ▶); software used to prepare material for publication: *OLEX2*.

## Supplementary Material

Crystal structure: contains datablocks I, global. DOI: 10.1107/S1600536811003102/su2246sup1.cif

Structure factors: contains datablocks I. DOI: 10.1107/S1600536811003102/su2246Isup2.hkl

Additional supplementary materials:  crystallographic information; 3D view; checkCIF report

## Figures and Tables

**Table 1:** Hydrogen-bond geometry (Å, °)

*D*—H⋯*A*	*D*—H	H⋯*A*	*D*⋯*A*	*D*—H⋯*A*
C13—H13⋯O6	0.93	2.60	3.222 (3)	125
C24—H24⋯O7^i^	0.93	2.41	3.327 (3)	170
C28—H28⋯O2^ii^	0.93	2.56	3.469 (3)	165
C30—H30⋯N23^iii^	0.93	2.62	3.489 (3)	155

Symmetry codes: (i) 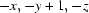; (ii) 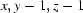; (iii) 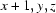.

**Fig. 1.**
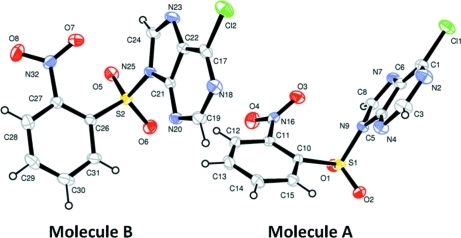
The molecular structure of the two independent molecules of the title compound, with displacement ellipsoids drawn at the 50% probability level.

**Fig. 2.**
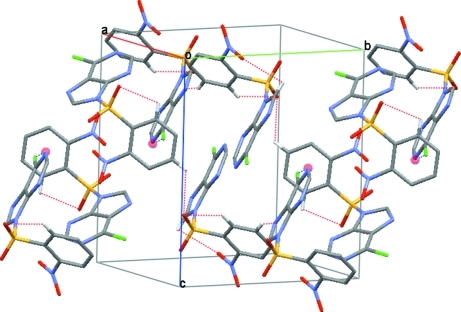
A view of the crystal packing of the title compound, with the C-Cl···π, C-H···O and C-H···N interactions shown as dotted red lines [the centroid of ring (C10-C15) is shown as a red dot].

## supplementary crystallographic information

### Comment

Purine derivatives are of great importance owing to their wide-ranging biological properties (Scozzafava *et al.*, 2001; Bakkestuen *et al.*, 2005). As there are several kinds of tautomers in purine derivatives, it is difficult to determine their structures by NMR, MS or IR sepctroscopy. The title compound is one of the key intermediates in our synthetic investigations of antimicrobial agents. Here we determined the accurate structure of the title compound by X-ray analysis.

As shown in Fig. 1, the title compound crystallized with two independent molecules (A and B) in the asymmetric unit. The conformation of the molecules is different. The benzene ring makes a dihedral angle of 66.46 (8)° with the mean plane of the purine ring in molecule A, while in molecule B this same angle is 85.77 (9)°.

In the crystal, the two molecules and symmetry related molecules, are linked into a three-dimensional network by intermolecular π···π stacking interactions involving ring (C10-C15) and a symmetry related ring (code: 1-x, 2-x, 1-z)], with a centroid-to-centroid distance of 3.8968 (12) Å, and nonclassical C—H···O and C—H···N hydrogen bonds (Table 1 and Fig. 2). There are also C-Cl···π interactions involving chlorine Cl2 and ring (C10-C15 = Cg), with a Cl···centroid distance of 3.2505 (10) Å, angle C17-Cl2···Cg^i^ being 161.56 (18)° [symmetry code: (i) -x, -y+1, -z+1] - see Fig. 1.

### Experimental

A mixture of 6-chloropurine (0.463 g, 3 mmol), 2-nitrobenzenesulfonyl chloride (1.33 g, 6 mmol), Triethylamine (0.607 g, 6 mmol), DMAP (0.037 g, 0.3 mmol), THF (10 ml) and DCM (10 ml) was stirred for 12 h at room temperiture. The solvent was removed under vacuum. The residue was extracted with ethyl acetate (50 ml) and water (50 ml). The organic layer was washed three times with 30 ml ammonia solution (5 N) and 30 ml brine, and then dried with anhydrous sodium sulfate. The product was isolated by column chromatography on silica gel. Yield 0.712 g (69.8%). Crystals, suitable for X-ray analysis, were obtained by slow evaporation from a solution of ethyl acetate.

### Refinement

All H atoms were positioned geometrically and refined using a riding model, with C—H = 0.93 Å and *U*_iso_(H) = 1.2*U*_eq_(C). As the centroid of the benzene ring holds partial positive charge and the chlorine atom at the purine ring holds partial negative charge, the chlorine atom in one molecular is likely to be close to the benzene ring of another molecular (see Comment section), leading to the nitro groups of two neighbouring molecules approaching one another. Hence, a short O3···O3^i^ distances [2.835 (2) Å] was observed in the crystal [symmetry code: (i) = -x, -y+2, -z+1)].

### Crystal data

**Table:**

C_11_H_6_ClN_5_O_4_S	*Z* = 4
*M**_r_* = 339.72	*F*(000) = 688
Triclinic, *P*1	*D*_x_ = 1.704 Mg m^−^^3^
Hall symbol: -P 1	Mo *K*α radiation, λ = 0.7107 Å
*a* = 10.0055 (3) Å	Cell parameters from 5646 reflections
*b* = 10.6931 (5) Å	θ = 3.1–29.1°
*c* = 12.5378 (5) Å	µ = 0.47 mm^−^^1^
α = 93.692 (3)°	*T* = 293 K
β = 97.136 (3)°	Block, colourless
γ = 93.995 (3)°	0.42 × 0.40 × 0.35 mm
*V* = 1324.16 (9) Å^3^	

### Data collection

**Table:**

Oxford Diffraction Xcalibur Eos diffractometer	5403 independent reflections
Radiation source: fine-focus sealed tube	4389 reflections with *I* > 2σ(*I*)
graphite	*R*_int_ = 0.018
Detector resolution: 16.0874 pixels mm^-1^	θ_max_ = 26.4°, θ_min_ = 3.1°
ω scans	*h* = −12→12
Absorption correction: multi-scan (*CrysAlis PRO*; Oxford Diffraction, 2006)	*k* = −13→13
*T*_min_ = 0.992, *T*_max_ = 1.0	*l* = −15→12
10984 measured reflections	

### Refinement

**Table:**

Refinement on *F*^2^	Primary atom site location: structure-invariant direct methods
Least-squares matrix: full	Secondary atom site location: difference Fourier map
*R*[*F*^2^ > 2σ(*F*^2^)] = 0.037	Hydrogen site location: inferred from neighbouring sites
*wR*(*F*^2^) = 0.092	H-atom parameters constrained
*S* = 1.02	*w* = 1/[σ^2^(*F*_o_^2^) + (0.0398*P*)^2^ + 0.4744*P*] where *P* = (*F*_o_^2^ + 2*F*_c_^2^)/3
5403 reflections	(Δ/σ)_max_ = 0.001
397 parameters	Δρ_max_ = 0.29 e Å^−^^3^
0 restraints	Δρ_min_ = −0.38 e Å^−^^3^

### Special details

**Table:**

Experimental. CrysAlisPro, Oxford Diffraction Ltd., Empirical absorption correction using spherical harmonics, implemented in SCALE3 ABSPACK scaling algorithm.
Geometry. All e.s.d.'s (except the e.s.d. in the dihedral angle between two l.s. planes) are estimated using the full covariance matrix. The cell e.s.d.'s are taken into account individually in the estimation of e.s.d.'s in distances, angles and torsion angles; correlations between e.s.d.'s in cell parameters are only used when they are defined by crystal symmetry. An approximate (isotropic) treatment of cell e.s.d.'s is used for estimating e.s.d.'s involving l.s. planes.
Refinement. Refinement of *F*^2^ against ALL reflections. The weighted *R*-factor *wR* and goodness of fit *S* are based on *F*^2^, conventional *R*-factors *R* are based on *F*, with *F* set to zero for negative *F*^2^. The threshold expression of *F*^2^ > σ(*F*^2^) is used only for calculating *R*-factors(gt) *etc*. and is not relevant to the choice of reflections for refinement. *R*-factors based on *F*^2^ are statistically about twice as large as those based on *F*, and *R*- factors based on ALL data will be even larger.

### Fractional atomic coordinates and isotropic or equivalent isotropic displacement parameters (Å^2^)

**Table:**

	*x*	*y*	*z*	*U*_iso_*/*U*_eq_	
Cl1	−0.28373 (6)	1.01998 (7)	0.86805 (5)	0.05386 (18)	
Cl2	−0.10359 (6)	0.30928 (6)	0.45966 (6)	0.05239 (17)	
S1	0.31769 (5)	1.10135 (5)	0.71476 (4)	0.02990 (13)	
S2	0.29292 (5)	0.60128 (5)	0.16093 (4)	0.03061 (13)	
O1	0.33387 (14)	1.21495 (14)	0.66297 (12)	0.0373 (3)	
O2	0.39441 (14)	1.08250 (15)	0.81453 (11)	0.0403 (4)	
O3	0.11604 (15)	1.08355 (17)	0.49868 (14)	0.0495 (4)	
O4	0.25354 (19)	1.11017 (18)	0.38100 (14)	0.0578 (5)	
O5	0.22219 (15)	0.63512 (15)	0.06322 (11)	0.0405 (4)	
O6	0.37133 (15)	0.69221 (15)	0.23407 (12)	0.0446 (4)	
O7	0.13521 (15)	0.38259 (17)	0.02911 (13)	0.0468 (4)	
O8	0.2366 (2)	0.3163 (2)	−0.10209 (14)	0.0737 (6)	
N2	−0.0813 (2)	0.8795 (2)	0.90380 (17)	0.0505 (5)	
N4	0.13616 (18)	0.90598 (18)	0.84106 (15)	0.0419 (5)	
N7	−0.05280 (17)	1.15618 (18)	0.74289 (14)	0.0375 (4)	
N9	0.15488 (16)	1.08978 (16)	0.73571 (13)	0.0308 (4)	
N16	0.21920 (18)	1.06359 (17)	0.46067 (14)	0.0369 (4)	
N18	0.15591 (18)	0.36990 (18)	0.50525 (15)	0.0402 (4)	
N20	0.30089 (16)	0.47424 (17)	0.39320 (13)	0.0335 (4)	
N23	−0.03515 (17)	0.4610 (2)	0.25421 (15)	0.0409 (5)	
N25	0.17329 (15)	0.53615 (17)	0.22797 (13)	0.0303 (4)	
N32	0.23641 (18)	0.35675 (18)	−0.00913 (14)	0.0387 (4)	
C1	−0.1216 (2)	0.9794 (2)	0.85674 (18)	0.0391 (5)	
C3	0.0440 (2)	0.8481 (2)	0.8933 (2)	0.0517 (6)	
H3	0.0702	0.7769	0.9267	0.062*	
C5	0.0901 (2)	1.0062 (2)	0.79701 (16)	0.0315 (4)	
C6	−0.0380 (2)	1.0496 (2)	0.79999 (16)	0.0331 (5)	
C8	0.0617 (2)	1.1767 (2)	0.70684 (17)	0.0358 (5)	
H8	0.0802	1.2434	0.6654	0.043*	
C10	0.33847 (18)	0.97337 (19)	0.62325 (15)	0.0287 (4)	
C11	0.30456 (19)	0.9715 (2)	0.51123 (16)	0.0310 (4)	
C12	0.3430 (2)	0.8782 (2)	0.44359 (17)	0.0372 (5)	
H12	0.3214	0.8790	0.3693	0.045*	
C13	0.4141 (2)	0.7834 (2)	0.48677 (19)	0.0406 (5)	
H13	0.4398	0.7196	0.4414	0.049*	
C14	0.4473 (2)	0.7827 (2)	0.59658 (19)	0.0413 (5)	
H14	0.4940	0.7177	0.6251	0.050*	
C15	0.4114 (2)	0.8783 (2)	0.66493 (18)	0.0361 (5)	
H15	0.4364	0.8785	0.7389	0.043*	
C17	0.0491 (2)	0.3758 (2)	0.43304 (18)	0.0356 (5)	
C19	0.2746 (2)	0.4186 (2)	0.48183 (18)	0.0396 (5)	
H19	0.3484	0.4132	0.5338	0.047*	
C21	0.18924 (19)	0.48116 (19)	0.32559 (15)	0.0282 (4)	
C22	0.05870 (19)	0.4349 (2)	0.33929 (17)	0.0326 (5)	
C24	0.0355 (2)	0.5197 (2)	0.19095 (17)	0.0393 (5)	
H24	−0.0021	0.5484	0.1263	0.047*	
C26	0.39753 (18)	0.4774 (2)	0.13725 (15)	0.0290 (4)	
C27	0.3671 (2)	0.3761 (2)	0.05959 (16)	0.0322 (5)	
C28	0.4589 (2)	0.2887 (2)	0.04330 (18)	0.0429 (5)	
H28	0.4371	0.2224	−0.0090	0.051*	
C29	0.5839 (2)	0.3008 (3)	0.1056 (2)	0.0478 (6)	
H29	0.6455	0.2410	0.0965	0.057*	
C30	0.6173 (2)	0.4008 (3)	0.1809 (2)	0.0495 (6)	
H30	0.7022	0.4093	0.2215	0.059*	
C31	0.5248 (2)	0.4892 (2)	0.19642 (17)	0.0398 (5)	
H31	0.5486	0.5570	0.2471	0.048*	

### Atomic displacement parameters (Å^2^)

**Table:**

	*U*^11^	*U*^22^	*U*^33^	*U*^12^	*U*^13^	*U*^23^
Cl1	0.0334 (3)	0.0680 (4)	0.0641 (4)	0.0061 (3)	0.0164 (3)	0.0127 (3)
Cl2	0.0409 (3)	0.0498 (4)	0.0706 (4)	−0.0040 (3)	0.0228 (3)	0.0140 (3)
S1	0.0250 (2)	0.0325 (3)	0.0309 (3)	0.0005 (2)	0.00197 (19)	−0.0021 (2)
S2	0.0309 (3)	0.0314 (3)	0.0304 (3)	0.0021 (2)	0.0060 (2)	0.0050 (2)
O1	0.0349 (8)	0.0312 (8)	0.0451 (9)	−0.0042 (6)	0.0067 (6)	0.0009 (7)
O2	0.0343 (8)	0.0509 (10)	0.0332 (8)	0.0074 (7)	−0.0038 (6)	−0.0041 (7)
O3	0.0367 (8)	0.0559 (11)	0.0569 (10)	0.0116 (8)	0.0042 (8)	0.0077 (9)
O4	0.0723 (12)	0.0616 (12)	0.0418 (10)	0.0071 (10)	0.0082 (9)	0.0178 (9)
O5	0.0444 (8)	0.0444 (9)	0.0360 (8)	0.0108 (7)	0.0079 (7)	0.0159 (7)
O6	0.0454 (9)	0.0379 (9)	0.0480 (9)	−0.0064 (7)	0.0069 (7)	−0.0073 (7)
O7	0.0319 (8)	0.0582 (11)	0.0482 (9)	0.0036 (8)	−0.0018 (7)	0.0009 (8)
O8	0.0699 (13)	0.1006 (17)	0.0438 (11)	0.0237 (12)	−0.0109 (9)	−0.0293 (11)
N2	0.0436 (11)	0.0514 (13)	0.0603 (13)	0.0045 (10)	0.0126 (10)	0.0212 (11)
N4	0.0382 (10)	0.0404 (11)	0.0492 (11)	0.0080 (9)	0.0064 (8)	0.0125 (9)
N7	0.0316 (9)	0.0404 (11)	0.0415 (10)	0.0076 (8)	0.0038 (8)	0.0086 (8)
N9	0.0275 (8)	0.0324 (10)	0.0333 (9)	0.0040 (7)	0.0050 (7)	0.0040 (7)
N16	0.0384 (10)	0.0354 (10)	0.0334 (10)	−0.0020 (8)	−0.0039 (8)	−0.0018 (8)
N18	0.0408 (10)	0.0407 (11)	0.0416 (11)	0.0053 (9)	0.0089 (8)	0.0131 (9)
N20	0.0270 (8)	0.0409 (11)	0.0328 (9)	0.0050 (8)	0.0018 (7)	0.0070 (8)
N23	0.0255 (9)	0.0576 (13)	0.0402 (11)	0.0048 (8)	0.0030 (8)	0.0080 (9)
N25	0.0247 (8)	0.0407 (10)	0.0267 (8)	0.0062 (7)	0.0036 (7)	0.0072 (7)
N32	0.0416 (10)	0.0380 (11)	0.0344 (10)	0.0059 (9)	−0.0034 (8)	−0.0013 (8)
C1	0.0314 (11)	0.0464 (14)	0.0392 (12)	0.0017 (10)	0.0040 (9)	0.0042 (10)
C3	0.0484 (14)	0.0478 (15)	0.0629 (17)	0.0086 (12)	0.0090 (12)	0.0244 (13)
C5	0.0313 (10)	0.0326 (11)	0.0300 (11)	0.0010 (9)	0.0025 (8)	0.0017 (9)
C6	0.0290 (10)	0.0370 (12)	0.0328 (11)	0.0028 (9)	0.0018 (8)	0.0038 (9)
C8	0.0333 (11)	0.0370 (12)	0.0380 (12)	0.0063 (9)	0.0034 (9)	0.0087 (10)
C10	0.0252 (9)	0.0301 (11)	0.0303 (10)	−0.0019 (8)	0.0056 (8)	−0.0009 (8)
C11	0.0254 (10)	0.0315 (11)	0.0349 (11)	−0.0024 (8)	0.0016 (8)	0.0019 (9)
C12	0.0353 (11)	0.0427 (13)	0.0323 (11)	−0.0029 (10)	0.0060 (9)	−0.0043 (10)
C13	0.0382 (11)	0.0382 (13)	0.0465 (13)	0.0031 (10)	0.0136 (10)	−0.0057 (10)
C14	0.0368 (11)	0.0366 (13)	0.0531 (14)	0.0093 (10)	0.0109 (10)	0.0058 (11)
C15	0.0347 (11)	0.0385 (12)	0.0360 (12)	0.0053 (9)	0.0056 (9)	0.0051 (10)
C17	0.0337 (11)	0.0309 (12)	0.0449 (13)	0.0022 (9)	0.0140 (10)	0.0067 (10)
C19	0.0353 (11)	0.0474 (14)	0.0367 (12)	0.0079 (10)	0.0009 (9)	0.0114 (10)
C21	0.0276 (10)	0.0286 (11)	0.0292 (10)	0.0050 (8)	0.0061 (8)	0.0012 (8)
C22	0.0266 (10)	0.0347 (12)	0.0374 (11)	0.0025 (9)	0.0068 (8)	0.0040 (9)
C24	0.0275 (10)	0.0553 (15)	0.0357 (12)	0.0113 (10)	−0.0001 (9)	0.0076 (11)
C26	0.0259 (9)	0.0358 (11)	0.0268 (10)	0.0029 (8)	0.0062 (8)	0.0083 (9)
C27	0.0313 (10)	0.0375 (12)	0.0288 (10)	0.0054 (9)	0.0034 (8)	0.0084 (9)
C28	0.0493 (13)	0.0434 (14)	0.0397 (13)	0.0146 (11)	0.0122 (10)	0.0063 (10)
C29	0.0393 (12)	0.0632 (17)	0.0484 (14)	0.0233 (12)	0.0163 (11)	0.0208 (13)
C30	0.0276 (11)	0.0752 (19)	0.0481 (14)	0.0081 (12)	0.0040 (10)	0.0210 (14)
C31	0.0281 (10)	0.0561 (15)	0.0346 (12)	−0.0015 (10)	0.0026 (9)	0.0070 (11)

### Geometric parameters (Å, °)

**Table:**

Cl1—C1	1.728 (2)	N25—C24	1.394 (3)
Cl2—C17	1.720 (2)	N32—C27	1.468 (3)
S1—O1	1.4226 (15)	C1—C6	1.378 (3)
S1—O2	1.4178 (15)	C3—H3	0.9300
S1—N9	1.6794 (16)	C5—C6	1.397 (3)
S1—C10	1.769 (2)	C8—H8	0.9300
S2—O5	1.4177 (15)	C10—C11	1.402 (3)
S2—O6	1.4150 (16)	C10—C15	1.385 (3)
S2—N25	1.6833 (16)	C11—C12	1.374 (3)
S2—C26	1.777 (2)	C12—H12	0.9300
O3—N16	1.216 (2)	C12—C13	1.379 (3)
O4—N16	1.221 (2)	C13—H13	0.9300
O7—N32	1.214 (2)	C13—C14	1.376 (3)
O8—N32	1.217 (2)	C14—H14	0.9300
N2—C1	1.317 (3)	C14—C15	1.387 (3)
N2—C3	1.340 (3)	C15—H15	0.9300
N4—C3	1.336 (3)	C17—C22	1.380 (3)
N4—C5	1.322 (3)	C19—H19	0.9300
N7—C6	1.391 (3)	C21—C22	1.399 (3)
N7—C8	1.293 (3)	C24—H24	0.9300
N9—C5	1.393 (3)	C26—C27	1.400 (3)
N9—C8	1.395 (3)	C26—C31	1.385 (3)
N16—C11	1.471 (3)	C27—C28	1.379 (3)
N18—C17	1.320 (3)	C28—H28	0.9300
N18—C19	1.337 (3)	C28—C29	1.382 (3)
N20—C19	1.339 (3)	C29—H29	0.9300
N20—C21	1.325 (2)	C29—C30	1.374 (4)
N23—C22	1.387 (3)	C30—H30	0.9300
N23—C24	1.290 (3)	C30—C31	1.390 (3)
N25—C21	1.388 (2)	C31—H31	0.9300
			
O1—S1—N9	104.73 (9)	C6—C1—Cl1	120.78 (17)
O1—S1—C10	108.86 (9)	C8—N7—C6	104.56 (17)
O2—S1—O1	121.88 (10)	C8—N9—S1	125.05 (14)
O2—S1—N9	106.39 (9)	C10—C11—N16	122.06 (18)
O2—S1—C10	107.43 (9)	C10—C15—C14	120.1 (2)
O3—N16—O4	124.65 (19)	C10—C15—H15	119.9
O3—N16—C11	117.33 (18)	C11—C10—S1	124.35 (16)
O4—N16—C11	117.93 (18)	C11—C12—H12	120.3
O5—S2—N25	105.10 (8)	C11—C12—C13	119.4 (2)
O5—S2—C26	111.24 (9)	C12—C11—N16	116.63 (19)
O6—S2—O5	121.33 (10)	C12—C11—C10	121.2 (2)
O6—S2—N25	106.67 (9)	C12—C13—H13	119.8
O6—S2—C26	106.90 (10)	C13—C12—H12	120.3
O7—N32—O8	123.88 (19)	C13—C14—H14	119.8
O7—N32—C27	118.70 (17)	C13—C14—C15	120.4 (2)
O8—N32—C27	117.42 (18)	C14—C13—C12	120.3 (2)
N2—C1—Cl1	117.83 (17)	C14—C13—H13	119.8
N2—C1—C6	121.4 (2)	C14—C15—H15	119.9
N2—C3—H3	115.9	C15—C10—S1	116.41 (15)
N4—C3—N2	128.3 (2)	C15—C10—C11	118.50 (19)
N4—C3—H3	115.9	C15—C14—H14	119.8
N4—C5—N9	128.60 (18)	C17—N18—C19	117.34 (18)
N4—C5—C6	126.78 (19)	C17—C22—N23	133.58 (18)
N7—C6—C5	111.24 (17)	C17—C22—C21	115.02 (18)
N7—C8—N9	113.85 (19)	C21—N20—C19	111.42 (17)
N7—C8—H8	123.1	C21—N25—S2	128.61 (13)
N9—S1—C10	106.61 (9)	C21—N25—C24	105.71 (16)
N9—C5—C6	104.60 (17)	C22—C17—Cl2	120.68 (17)
N9—C8—H8	123.1	C24—N23—C22	104.34 (17)
N18—C17—Cl2	118.10 (16)	C24—N25—S2	125.56 (14)
N18—C17—C22	121.22 (18)	C26—C27—N32	122.17 (18)
N18—C19—N20	128.4 (2)	C26—C31—C30	120.7 (2)
N18—C19—H19	115.8	C26—C31—H31	119.7
N20—C19—H19	115.8	C27—C26—S2	126.17 (15)
N20—C21—N25	128.98 (17)	C27—C28—H28	120.3
N20—C21—C22	126.52 (18)	C27—C28—C29	119.3 (2)
N23—C22—C21	111.38 (17)	C28—C27—N32	116.2 (2)
N23—C24—N25	114.08 (18)	C28—C27—C26	121.6 (2)
N23—C24—H24	123.0	C28—C29—H29	119.9
N25—S2—C26	104.22 (9)	C29—C28—H28	120.3
N25—C21—C22	104.48 (16)	C29—C30—H30	119.9
N25—C24—H24	123.0	C29—C30—C31	120.3 (2)
C1—N2—C3	117.3 (2)	C30—C29—C28	120.2 (2)
C1—C6—N7	133.91 (19)	C30—C29—H29	119.9
C1—C6—C5	114.85 (19)	C30—C31—H31	119.7
C5—N4—C3	111.40 (19)	C31—C26—S2	115.78 (17)
C5—N9—S1	128.59 (14)	C31—C26—C27	117.84 (19)
C5—N9—C8	105.75 (16)	C31—C30—H30	119.9

### Hydrogen-bond geometry (Å, °)

**Table:**

*D*—H···*A*	*D*—H	H···*A*	*D*···*A*	*D*—H···*A*
C13—H13···O6	0.93	2.60	3.222 (3)	125
C15—H15···O2	0.93	2.41	2.814 (3)	106
C24—H24···O7^i^	0.93	2.41	3.327 (3)	170
C28—H28···O2^ii^	0.93	2.56	3.469 (3)	165
C30—H30···N23^iii^	0.93	2.62	3.489 (3)	155
C31—H31···O6	0.93	2.36	2.794 (3)	108

Symmetry codes: (i) −*x*, −*y*+1, −*z*; (ii) *x*, *y*−1, *z*−1; (iii) *x*+1, *y*, *z*.
